# Unboxing the black box—one step forward to understand the soil microbiome: A systematic review

**DOI:** 10.1007/s00248-022-01962-5

**Published:** 2022-02-02

**Authors:** Apurva Mishra, Lal Singh, Dharmesh Singh

**Affiliations:** 1 Academy of Scientific and Innovative Research [AcSIR], Ghaziabad, 201002 India; 2 Environmental Biotechnology and Genomics Division, , CSIR-National Environmental Engineering Research Institute, Nehru Marg, Nagpur, 440020 Maharashtra India; 3 Institute for Medical Microbiology, Immunology and Hygiene, Technical University of Munich, Trogerstrasse 30, 81675 Munich, Bavaria, Germany

**Keywords:** Soil microbiome, Nutrient cycling, Soil biodiversity, Soil networks, Climate change

## Abstract

Soil is one of the most important assets of the planet Earth, responsible for maintaining the biodiversity and managing the ecosystem services for both managed and natural ecosystems. It encompasses large proportion of microscopic biodiversity, including prokaryotes and the microscopic eukaryotes. Soil microbiome is critical in managing the soil functions, but their activities have diminutive recognition in few systems like desert land and forest ecosystems. Soil microbiome is highly dependent on abiotic and biotic factors like pH, carbon content, soil structure, texture, and vegetation, but it can notably vary with ecosystems and the respective inhabitants. Thus, unboxing this black box is essential to comprehend the basic components adding to the soil systems and supported ecosystem services. Recent advancements in the field of molecular microbial ecology have delivered commanding tools to examine this genetic trove of soil biodiversity. Objective of this review is to provide a critical evaluation of the work on the soil microbiome, especially since the advent of the NGS techniques. The review also focuses on advances in our understanding of soil communities, their interactions, and functional capabilities along with understanding their role in maneuvering the biogeochemical cycle while underlining and tapping the unprecedented metagenomics data to infer the ecological attributes of yet undiscovered soil microbiome. This review focuses key research directions that could shape the future of basic and applied research into the soil microbiome. This review has led us to understand that it is difficult to generalize that soil microbiome plays a substantiated role in shaping the soil networks and it is indeed a vital resource for sustaining the ecosystem functioning. Exploring soil microbiome will help in unlocking their roles in various soil network. It could be resourceful in exploring and forecasting its impacts on soil systems and for dealing with alleviating problems like rapid climate change.

**Supplementary Information:**

The online version contains supplementary material available at 10.1007/s00248-022-01962-5.

## Introduction

Soil is the most substantial pool of biological diversity and organic matter on terrestrial land that reinforces a huge range of processes important for life sustenance on Earth [28, 102]. It is still conceivably the least comprehended and acknowledged of Earth's environment. Soil accounts for denser biodiversity per unit territory than observed anywhere aboveground and it is a hub for biological and molecular interactions [6, 139]. With their subterranean networks, soil is firmly connected to aboveground networks through multi-trophic communications, nutrient cycling, and plant–soil feedback. It is also responsible for administering the functioning of ecosystems and working toward ecosystem services [7, 36]. Soil microscopic biomass matches or even in some cases exceeds the aboveground biomass, with soil frequently containing greater than a thousand kilograms of microscopic biomass carbon per hectare [9, 25]. The scientific world and the common man are more concerned about the macroscopic environment, and much lesser is known about the microscopic biota that is hidden in the heterogeneous soil, although these organisms along with plants, rule the Earth’s living biomass [9, 46]. Understanding the composition of soil microscopic biota and their functional role is increasing gradually with the advancement in sequencing techniques [46]. With this progressive understanding of soil microscopic biodiversity, it is required to investigate a variety of soil functions supported by an unknown catalogue of organisms that have a strong involvement in soil functions, along with other ecosystem services [13, 122]. The soil microbiome is also involved in climate change processes and regulation of greenhouse gas emissions [46, 100]. The recent soil microbiome insights with huge prospects are anticipated from all fields of life.

**Fig. 1:**
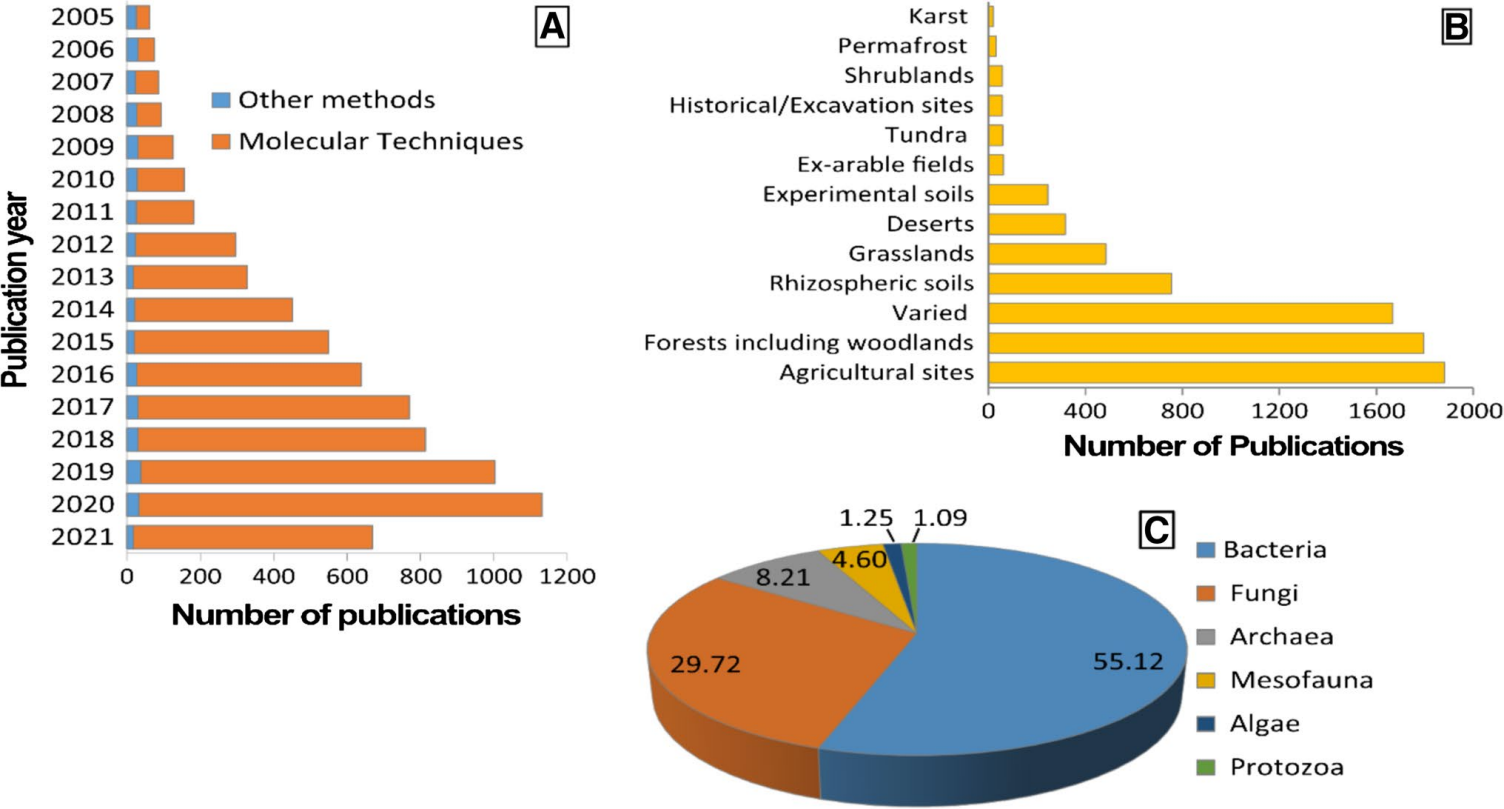
**Publication trend of scientific articles in the field of soil microbiome over time [A]** Numbers of scientific articles published in the field of the soil microbiome since advent of next generation sequencing in 2005. Molecular techniques include FISH, qPCR, DGGE, RFLP, RAPD, amplicon-based next-generation sequencing, and shotgun metagenomics. Other methods include BIOLOG, PLFA, CLPP, FAME, and methods like Oostenbrick extraction, sugar flotation, Baermann funnel, and Tullgrens method followed by microscopy and enumeration **[B]** Number of scientific articles published under different terrestrial; natural or man-made ecosystems throughout the years. Agricultural sites include plantations, vineyards, orchards, arable lands, farmlands, and pastures; experimental soil including greenhouse or laboratory experiments, forensics soil samples, landfills, ore mines, etc.; rhizospheric soil include rhizosphere, rhizoplane and phyllosphere [most of the studies featuring rhizospheric soil also studied bulk soil]; deserts including arid or semiarid regions; grasslands including savannahs, meadows, etc.; varied includes publications studying more than two different sites in a single study. **[C]** Numbers of scientific articles published in the field of the soil microbiome distributed on the basis of the organism studied

To decipher the current state of knowledge available for soil microbiome and understanding the trend in soil microbiome study, we performed a systematic literature search using the Clarivate Web of Science Core Collection on 22.7.2021 following with combination of keywords as—*soil* AND microbiom* OR *soil* AND biota* OR *soil* AND *biodiversit* ranging from a period of 2005 to 2021. This time period was chosen due to introduction of sequencing-by-synthesis technology was introduced by 454 Life Sciences in 2005, [90]. This technology eventually revolutionized the field of soil microbiome in years to follow and continues to do so. To narrow it down, document types under book chapters, editorial managements, reviews, books, news items, book reviews, reprints, retracted publications, biographical items, and retractions were removed from the searched results, yielding 21,745 studies. This list was further screened manually to remove ones focusing only on culturable microbial diversity or the ones studying only soil macro-invertebrates or plants or unrelated topics etc. This led to a final list containing 7434 studies and now we could say with authority that the scientific community has started to take more and more interest into the subject of soil microbiome in the recent years Fig. 1.

The presence and role of soil microbiome remains understated in soil processes, which could be possibly due to the notion of it being ecologically redundant [36]. Many basic physiological and behavioral aspects of soil organisms; their interactive biotic relationships among themselves or with the above soil biota; their responses to the different abiotic and biotic factors; their spatial and temporal patterns and functional roles concerning soil niche—are regularly ignored or less explored as compared to the above ground biodiversity [46]. The aim of this review is to provide a critical evaluation of the work on the soil microbiome, especially since the advent of the NGS techniques. The novelty of this review lies in the systemic review of literature used above, wherein the details of tools used are explored over the period of time to discover the unexplored entity. The review also focuses on potential threats to soil microbiome. It also talks about the impact of climate change on the soil microbiome that is one of the urgent topic of the present times. This review is a sincere effort to combine the information about the current status of soil microscopic biome comprehensively and holistically, coupling soil assembly and biological processes with the current experimental and theoretical knowledge to improve predictive capacities. This improved understanding of soil biodiversity could be resourceful to supply plausible answers for the reported soil microbiome responses to environmental deviation like climate change. It is necessary to recognize and understand the functioning of this microscopic biome to build sustainable management of the soil and enrich the ecosystem along with its services.

## Factors affecting soil microbiome

The richness and diversity of the soil microbiome are coordinated with the functioning of ecosystems and its composition within the soil environment relies on both chemical and physical properties of soil along with the anthropogenic factors that affect them [36]. The range of soil conditions affecting the organisms inhabiting are called edaphic factors, and they are part of abiotic factors.

The structure of the soil microbiome is affected largely by the edaphic properties and is the basic ecological filter [24]. Factors like soil structure influences the development of mesofauna, due to the high number of micropores in clay molecules, which in turn hampers the predatory activity of microorganisms. Similarly, distinctive types of soil biota have their very own inclinations for pH and it is observed that soil pH is significantly correlated with the development of a specific set of the soil microbiome. For instance, liming of acidic soil often results in shifts in the composition and abundance of soil microbiome including bacteria, fungi, archaea, nematodes, annelids, and microarthropods, as there is a significant increase in pH and nutrient concentrations [56]. With an increase in pH, the soil bacterial-to-fungal ratio and nematode abundance increase as well [142]. Liming does not significantly influence the diversity of mites and collembolans, but a slight variation in the species composition speaks about the increase in dependent species and subsequently decreases in abundances [18]. Protists, on the other hand, which span the majority of microscopic eukaryotes, are only marginally influenced by pH [10].

**Fig. 2:**
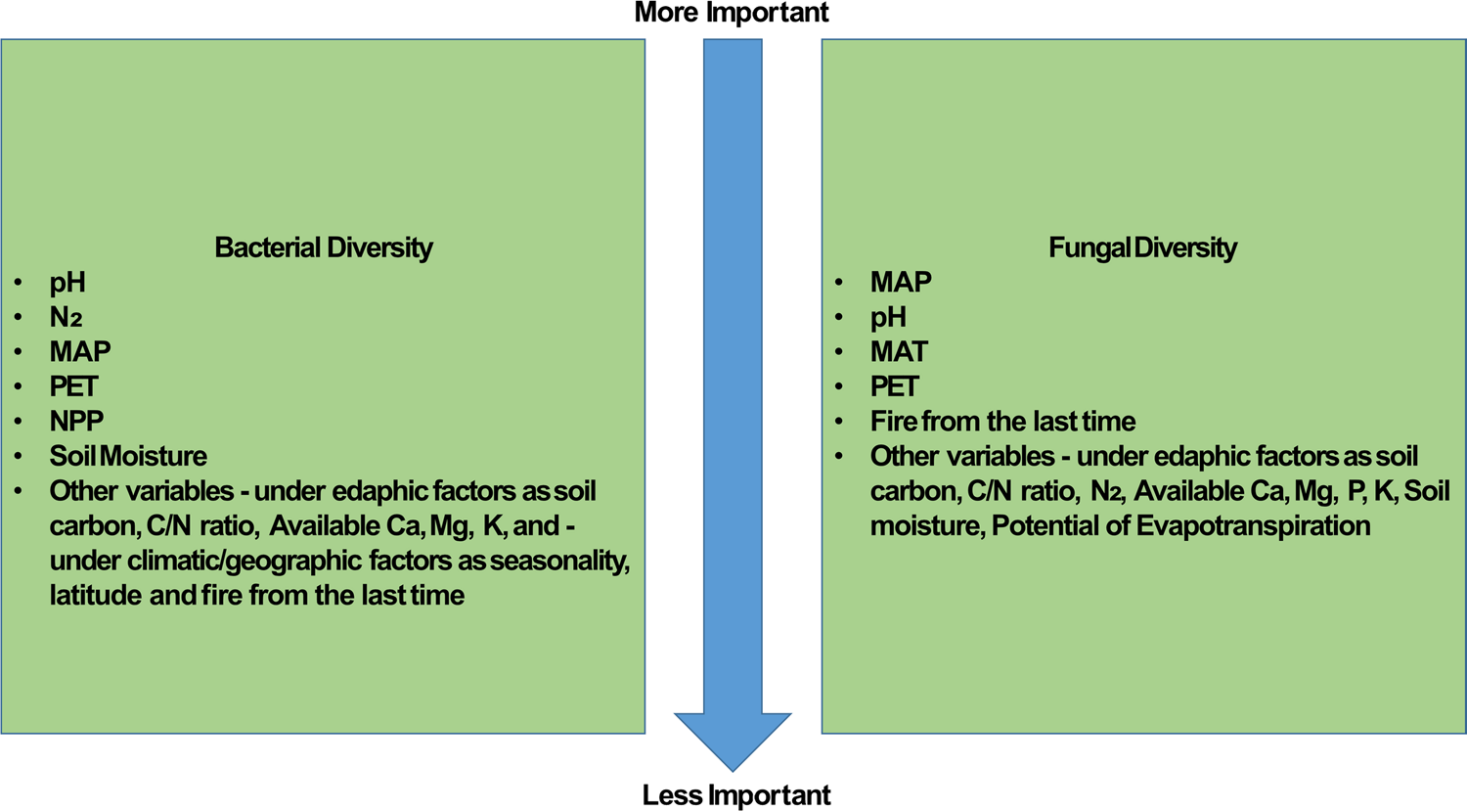
**Abiotic and biotic factors affecting soil bacterial and fungal communities.** The scale here is based on a global study by Bahram et al., 2018 https://doi.org/10.1038/s41586-018-0386-6 where they used metagenomics and metabarcoding to study topsoil samples [189 sites, 7,560 subsamples] for bacterial and fungal distribution and abundance on a latitudinal gradient. Abbreviations: MAT-mean annual temperature; MAP-mean annual precipitation; PET- potential evapotranspiration; NPP-net primary production

Bacterial phyla such as Actinobacteria, Gemmatimonadetes, β and δ-Proteobacteria, Chloroflexi, and Nitrospirae strongly correlate with soil pH and favor distinct optimal pH, which eventually shapes the microbial structure and diversity in soil along with the other factors as shown in Fig. 2 [7]. For bacteria, edaphic factors like soil pH and other soil nutrient concentrations strongly regulate its taxonomic diversity and composition due to the facilitation of nutrient accessibility in the soil, whereas in the case of fungi, climatic variables such as mean annual precipitation seem to have an upper hand over pH when explaining the diversity [7]. As change in climatic condition, like when CO_2_ is elevated enhances the abundance and activity of mycorrhizal fungi in relation to the production of spore-bearing structures, the fungal abundance increases.

The moisture content affects the microbiome as water availability is very important for maintaining the microbial life and helps in their mobility in soil, dissolution of nutrients, and diffusion of gases [153]. Soil moisture was found to be influential in shaping the protistan community composition. Soil type and structure also affect the community composition of the soil microbiome with lighter soil structure favoring the growth of bacteria [41, 128]. Soil with clay molecules and a large number of micropores are found to limit the growth of mesofauna, which protect the microbes from predation [94]. Soil nutrient concentration such as carbon content and C/N ratio plays a pivotal role in managing the diversity and structure of the soil microbiome [114, 157].

### The functional role of soil microbiome

Soil microbiome plays a varied role in the terrestrial environments and understanding these can assist us in predicting the range of impacts, they may have on both natural and managed ecosystems Table 1. Soil microbiome is answerable for the mineralization of organic matter and nutrient discharge [61, 139] with groups like protozoans and nematodes assuming an essential job in the predation of soil microflora. These expand microbial respiration and stimulate nutrient mineralization like phosphorus [68]. The litter decomposition rate is chiefly directed by climatic conditions, i.e., temperature and precipitation, at large spatial scales, while at a minor spatial scale, nutrient contents are more important [93]. However, eventually, soil organic matter [SOM] and litter disintegration are ruled by biotic activities [72]. There are numerous functional jobs that soil microbiome performs while balancing the ecosystem as described in Table 1 and sub-sections below.

**Table 1:** Functions performed by soil microbiome

Organisms	Functional Group	Representative Member/s	Major Functions	References
**Mesofauna**	Soil invertebrates	Collembola, Enchytraeids	Aboveground shredding of litter Development of soil structure Decomposition and carbon cycling	[42, 95]
**Mainly bacteria and fungi**	Decomposers	Mycorrhizal Fungi, β-proteobacteria	Retain nutrients in their biomes Synthesis of SOM Decomposition of SOM Carbon sequestration Emissions of CO_2_ and CH_4_ Enhance plant growth Promote disease	[50, 55, 63, 66, 100]
**Heterotrophic and autotrophic bacteria, fungi**	Nutrients transformers	Nitrosomonas, Pseudomonas, Endophytes Cyanobacteria	Nitrification and denitrification Phosphorous solubilization Nutrient cycling Add organic matter to soil Nitrogen fixation Soil stabilizers Microbial residues stabilizer Breakdown of nutrients	[29, 82, 111, 145]
**Nitrogen-fixing bacteria**	Nitrogen fixers	Rhizobium	Converting atmospheric N to amides and ammonium ions Nitrogen fixation	[84]
**Meiofauna**	Biological Indicators	Nematodes, Protozoans	Decomposition of organic matter Nutrient cycling Soil quality Stimulate and control the activity of bacterial populations Control many disease-causing pests	[83, 107]
**Microscopic Eukaryotes**	Bio-indicators	Protists	Energy transfer Key job as being consumer feeding bacteria, fungi and other small eukaryotes in food web Improving soil quality	[10, 45]
	Soil Mineralization	Protozoa	Regulate bacterial populations Release plant available nitrogen and other nutrients when feeding on bacteria	[59]
**Archaea**	Soil nitrifiers Nutrient Transformers	Ammonia oxidizing archaea Crenarchaeota	Soil nitrification Ammonia oxidation Carbon and nitrogen turnover Nitrification process	[59, 151]

### Role of the soil microbiome in managing SOM and C sequestration

Soil microbiome with plant diversity is a huge participant in carbon storage and sequestration; they serve plentiful roles in the pedosphere, critical for the formation and turnover of SOM [130] to fulfil their needs of nutrient and energy for their growth and maintenance [81]. The prime way that C is stored in soil is in the form of soil organic matter [SOM] with around 80% of C in stable fractions of SOM is constituted of microbial residues and exudates [132, 147]. It shows that soil microbial biomass [SMB] holds a binary role in SOM turnover, balancing SOM mineralization and stabilization. A diverse range of organisms underwrite significantly to the living microbial mass of SOM [98], and the rate of degradation of organic matter is controlled by the microbiome and the abiotic factors like soil pH and temperature and in turn controls the microbial activity [149]. SOC storage is also affected by soil microbiome, influencing the soil structures, e.g., the burrowing activity of earthworms and enchytraeids is changed due to soil macro-porosity [33, 62, 116]. Other than earthworms, millipedes [136, 148], soil meiofauna such as mites, springtails, and enchytraeids are found to protect the OM from soil aggregation via fecal pellets which hold a huge amount of C in soil ecosystems [134, 135]. Predation by protists leads to a decline in the total bacterial biomass [30], an increase in the nutrient turnover, and overall stimulation of the soil microbiome [43]. Prokaryotic components of the microbiome like archaea carry out the two essential metabolisms for nutrient cycling globally, i.e., methanogenesis- and sulfate-dependent anaerobic methane oxidation [106]. In conclusion, the soil microbiome plays a huge role in the functioning of the ecosystem and performs different activities in partial fulfilment of it as discussed in Table 1 that mentions the representative taxa and the type of functions they perform.

### Role of the soil microbiome in nutrient cycling

In the soil ecosystem, the nutrients are involved in a myriad of chemical and biochemical reactions, which are largely mediated by the soil microbiome. Soil microorganisms are acknowledged to support a plethora of functions related to carbon [C] cycling [140]. The balance between respiration and photosynthesis is dominant in C cycles on terrestrial systems. The atmospheric C in form of CO_2_ is fixed in soil by photo and chemoautotrophic microbes through Calvin cycle with the key enzyme ribulose 1,5-bisphosphate carboxylase/oxygenase or RuBisCO, [154] Phototrophic microbes from α- β- and γ-Proteobacteria and Cyanobacteria actively fix atmospheric C from the soil using the Calvin Cycle [143]. Chemoautotrophic members from bacterial phyla such as α- Proteobacteria, Actinobacteria, and Chloroflexi have been actively seen to fix the atmospheric CO_2_ in atmospheres like grasslands under dark conditions [143, 151], whereas chemoautrophic methanogenic archaea, acetogenes [86], can fix the atmospheric CO_2_ with inputs of exogenous H_2_ in paddy fields. In the soil C cycle, the role of mycorrhizal fungi is also important ranging from obligate symbionts like [AMF] that can solitary obtain carbon from the host plant to facultative symbionts like the ectomycorrhizal fungi [ECM] that can also mineralize organic carbon. Soil microbiome indisputably impacts the C cycle due to acts like the decomposition of litter, wherein the surface area increases. This allows the microbes to colonize more [52], followed by gathering of organic matter and stimulation of microbial activities [52]. Another important biogeochemical cycle is nitrogen cycle, where bacteria, archaea, and fungi help in converting the organic nitrogen into ammonium form by attacking the decaying plants and animals, i.e., the first step in the nitrogen cycle termed as ammonification [109]. After ammonification, specialized group of bacteria, and archaea, take over the chemical processes, like ammonia-oxidizing bacteria [AOB] and ammonia-oxidizing archaea [AOA], runs the nitrification process, thereby converting ammonia to nitrites and nitrates. β- and γ- Proteobacteria, Nitrospirae, and members of phylum Thaumarchaeota possess representative microbes involved in the process of nitrification [138]. On the contrary, few soil prokaryotes like diazotrophs have an enzyme called dinitrogenase, which converts soil inorganic and organic nitrogen into ammonia, followed by nitrite and nitrate [34].

Another major growth-limiting nutrient is phosphorus. Soil phosphorus is washed off to the environment via different means like runoff or leaching, resulting in agricultural issues mainly delay in crop maturity, the decline in seed quality, and low crop yield, due to insufficient soil P, and this is due to mineralization by soil microbes like bacteria [67]. Soil microbes like bacteria, archaea, and fungi help in solubilization and mineralization of P. Protozoans, nematodes, and other mesofauna help in immobilization of this available P and convert them to organic form [121]. AMF is also efficient in P uptake from soil and delivering it to plants [15].

### Threats to soil microbiome and biodiversity

As of now, soil and their biomes are being undermined by debasement brought about by global environmental and climatic changes. The condition is again worsened by anthropogenic activities including changes in land use, pollution, and the introduction of new invasive species in newer environments, with possibly far-reaching impacts on Earth's biological systems [27]. One of the biggest threats is human intervention. Since the inception of agriculture, humans have rehabilitated the plant's local diversity by clearing land and growing selected plant species [27]. Additionally, pollutants entering the soil as a result of activities like oil spills and mining are also influencing the soil microbiome and the enormous ecosystem functions, offered by the soil [152]. Additionally, this belowground diversity can also be heavily impacted by physical disturbances of soil like compaction, erosion, sealing, habitat fragmentation, and other activities.

In recent decades, worldwide climate change has globally modified precipitation and temperature systems, which is affecting soil biodiversity both directly and indirectly through their influence on plant diversity and primary productivity [150]. Attention to soil biodiversity and its utilitarian significance will empower the advancement of economical administration practices, for which we will need a better understanding of the possible threats to soil biodiversity. Listed below are a few possible threats to the soil biodiversity –

### Invasion of alien or exotic species

In natural ecosystems, different species have evolved together in such a way that usually no single species can overpower the system and hence they coexist. Then, there are invasive species, which are well known to pose risk to species diversity globally, and can be one of the reasons responsible for endemic biodiversity losses [118]. Biological invasion by alien species can have a remarkable impact on soil biodiversity and are of two types- invasive plants and invasion of the soil microbiome. Recent examinations have suggested that invasion by alien plants generally modifies the soil microbiome [155]. Although the effects vary with individual invasive plants and can have their distinct effect on the soil chemistry and structure, there could be cases where an invasive plant species with comparatively higher root biomass than local plants can release more nutrients and organic carbon to the surrounding rhizosphere. This eventually provides a stage for local soil microbes to flourish and can initiate positive feedback that promotes the invasive species. To cite one example*, Prosopis juliflora*, the Latin American tree which is tolerant to high temperatures and salinity stress, invaded semi-arid locations of Africa producing organic substances that are found to be toxic to the native plants due to allelopathic effects [69, 89]. To add upon, it can host native nitrogen-fixing bacteria, like *Rhizobium* and *Bradyrhizobium* bacteria once the conditions further improved**.** Another example of plant invasion is by the weed *Conyza canadensis,* which had contrasting effects on different groups of soil fungi. A decrease in abundance of Ascomycota and Chytridomycota was observed in conjunction with an increase in the numbers of phylum Glomeromycota [155] at different degrees of invasiveness by the weed. Invasive plants also considerably influence the catabolic diversity of the soil microbes via their effect on the soil enzymatic activities, which illustrates the connection between litter decomposition, availability of nutrients, and rhizospheric microbial activity [31, 73] but each invasive plant species will have their own idiosyncratic effects on the soil microbiome not reproducible by other aliens [47].

Organisms like ants and soil-dwelling flatworms, or fungal pathogens can also invade the soil microbiome. The intrusion of outsider’s microbiome in the new environment can meticulously affect their hosts and beneficiary ecosystems [48, 120]. Invasion of soil microbiome particularly with ecosystem engineers like earthworms can have a dramatic impact on the recipient ecosystems. They can lead to a significant decline in native soil invertebrate diversity like in collembolans and oribatid mites, significant decline in mycorrhizal fungal colonization as well as homogenization of the microbial communities across the soil layers by bioturbation which was observed [35].

The biological systems generally inclined to serious effects of invasive species are those that have been separated geographically for an exceptionally prolonged period. For example, islands, because their local species can be highly different from the exotic species. One such example is invasions of rats on off-shore islands in New Zealand, which lead to extensive and cascading effects on island soil saprophytic communities accompanied by significant decrease in populations of enchytraeids, bacterivore nematodes, rotifers, and collembolans [40]. Such results exhibit that alien species as predators can indirectly induce strong shifts in soil microbiome through disruption in several trophic levels and local interaction pathways in soil.

### Pollution

Soil is a tremendously complex environment that provides food, water, shelter, and air to the organisms inhabiting. Due to this property, pollutants that end up in the soil can have immediate effects on soil biodiversity [44]. Soil pollutants that are commonly seen are pesticides, heavy metals, high salt concentrations, oils, and fertilizers. Pollutants at a large range can end up in soil via varied routes like atmospheric fall, through waste disposal, industrial activities, accidental oil spills, and can have a far-reaching impact on the soil functioning.

Widespread pollutants as heavy metals [metal with a density ≥ of 5 g cm^−3^] are linked to toxicity and pollution. Heavy metals like copper, iron, and zinc are important for the physiological functioning of microbes to a given limit or else they become toxic, when present in high concentrations and their contamination leads to a decline in microbial biomass while enriching the heavy metal resistant microbial populations [97]. Amongst these, copper is reported to be one of the most common soil contaminants that can alter the community structure of many organisms like bacteria, archaea, and fungi [20] as it affects the various cellular enzymes and the proteins that are involved in the energy metabolism [144].

Agrochemicals like herbicides, insecticides, and chemical fertilizers are often used prudently, but still found to adversely affect the soil microbiome [92]. These chemicals are often applied to the field directly or sprayed on the soil strata and are hazardous for the non-targeted soil-borne organisms, which are assumed beneficial or commensal [103, 110]. Over 95% of the applied herbicides and 98% of insecticides influence the non-targeted soil organisms and soil, which unfavorably affects the soil microcosms involving, soil microscopic fauna in field networks and soil biological system [58, 60, 85]. Correspondingly, a few investigations have stated that various pesticides quell soil enzymatic activities, which ultimately influence the nutrient status of soil.

### Acid rain and overloading of nutrients

Acid rain is a mixture of both wet and dry material deposition from the atmosphere containing a higher amount of sulfuric and nitric acids. It mainly works by increasing soil acidity, affecting the microbes like bacteria and fungi, which further disrupt the organic matter transformed into nutrient forms. Among mesofauna, Collembolans and mites are sensitive to high acidity [146], while others such as enchytraeids are more resilient to high acidity. Communities of earthworms especially like *Eisenia fetida* are also affected by the soil acidification because earthworm do not survive at very acidic pH; instead, they need a continuous calcium supply. The change in pH is inversely proportional to the concentration of ionized calcium [75]. By directly decreasing the soil pH, acid rain helps in increasing the count of acid-tolerant soil fauna and vice versa. This in totality alters the soil microbiome [146] in conjunction with an increase in the soil bulk density thereby decreasing the movability and availability of H_2_O/O_2_, which further modifies the soil microbiome and affects the soil's ecological function and processes. Acid rain also has negative effects on the plants, e.g., by increasing the soil acidity, aluminum is allowed to get solubilized and its free organic form which is toxic to the plant roots, which subsequently locks up phosphate and meanwhile starts reducing the concentrations of the essential plant nutrients. However, it is seen that in such case, ectomycorrhizal fungal species present on the root surfaces of some trees help them with the supply of soluble calcium to the soil that are subjected to acid rains [108].

Soil globally receive nutrients in different ways, either through natural processes or human activities, although human activities produced a large number of nutrients, e.g., P and N_2_ inputs are exceptionally high via agricultural lands due to the application of fertilizers. A significant plant community shift is observed over time in non-agricultural soil due to the excessive addition of nutrients. Nutrient flooding leads to change in the soil pH and shifts the carbon dynamics belowground due to changes in amounts and types of organic carbon, derived from plants and litter that are mineralized to carbon dioxides via microbial activities [108]. When soil are N_2_ or P amended or overloaded, the abundance of mycorrhizal fungal taxa usually declines and the growth of cyanobacterial taxa is inhibited [108]. One of the direct impacts of nutrient overloading on soil microbiome includes a reduction in the relative abundance of nitrogen-fixing bacteria and an increase in the taxa responsible for denitrification or nitrification process [108]. In general, addition of nutrients tends to encourage the taxa that have high demands for N_2_ and P and shift bacterial and fungal communities due to these cascading effects, thereby affecting the soil food web. This elevated nutrient soil profile is also known to impact the soil C cycle as it is generally observed that the decomposition of organic matter via microbes is lowered down when nitrogen is used as an amendment in soil. This is accompanied with a decline in the size of soil microbial biomass; however, the responses differ throughout different sites.

Given the myriad connections among plants and subterranean biota, one of the essential mechanisms through which nutrient alterations impact soil microbiome is by changing plant production and plant network types [113]. For instance, the addition of nutrients often supports the growth of weedy plants, which is one of the producers of high-quality litter, inhibiting the growth of microbial taxa that are specialized in the decomposition of recalcitrant litter affecting the soil micro and mesofauna in various ways.

### Intensive agriculture practices

Effects of agriculture on soil microbiome are highly variable depending on the management system adopted. For instance, there are two types of agricultural systems—low input system or farming utilizes internal resources and tries to minimize the use of production inputs like purchased organic fertilizers and other is high input systems and utilizes huge equipment and commercial fertilizers, mostly for monoculture plantations using the conventional pattern often known as farming systems which utilize the synthetic fertilizers [11, 78]. A significant reduction in soil community diversity is generally observed in highly managed systems, while the diversity is mostly conserved in the lower input systems [37]. For example, the soil microfaunal, mesofaunal, and fungal community diversity is severely affected by the conversion of native forests to rubber monoculture plantations [133, 137]. Monocultures as rubber, palm, or even maize are generally accompanied by a progressive increase in soil bulk density and suffer from soil compaction in the top layer of soil. This affects the microbial community, which in turn were to be predated upon by soil micro- and mesofauna like protists and nematodes. Physical disturbance in soil due to compaction reduces niche availability for several microarthropods, annelids, and others soil dwellers, and this might further reduce the soil microbiome abundances [79, 99]. Monocultures seem to favor fast-growing fungal phyla like Ascomycota, and slow-growing fungal groups belonging to Basidiomycota seem to be adversely affected by physiochemical changes in the monocultures. As with these high input systems, there is more soil disturbance, soil management practices dictate removal of leaf litter and roots, and regular application of fertilizers and pesticide might cause a perturbation of soil, negatively affecting slow growers like Basidiomycota [137]. Bacterial pathways are more favored in the high input systems ruled by the opportunistic bacterial feeding nematodes; on the contrary, low-input systems indulge fungal pathways dominated by the fungal feeding fauna-like termites. Protists are also negatively affected by the conversion of the rain forest to other plantations, whereas there is an increase in bacterial diversity [12, 124, 127]. Abundance of root-associated microbiota such as arbuscular mycorrhizal fungi [AM] as well as their network connectivity tends to be drastically affected by intense agricultural practices and is more preserved under low input systems such as organic farming [8].

A significant alteration in the soil, especially linked to soil structure, soil organic content, water-holding capacity, and porosity, causes soil tillage. The influence of tillage on the soil microbiome is highly variable, dependent on the soil characteristics and the type of tillage system [80]. There are three main tillage systems known- conventional tillage, i.e., plowing that breaks down the soil thereby destroying the soil structure and burying the crop residues, causing the most significant impact on soil-dwelling organisms [125]. The second type is the minimum tillage system, i.e., characterized by a reduction in tillage area and the last one is the no-tillage system, where the soil surface is relatively undisturbed and providing a comparatively stable habitat. It is seen that bacteria are more favored by conventional tillage systems, which in turn expect protists also to be favored as bacteria are their main source of food [156]. Collembolans are generally inhibited due to disturbances created by tillage depending on the type of environment and contrary mites showcase a wide range and more severe responses [16, 21, 70]. A study revealed that intensive agriculture is responsible for a reduction in soil biodiversity across Europe. In the study, four agricultural regions across Europe were examined for the effect of land-use intensity formulated based on structural diversity among the groups along with the body mass of the soil fauna. It was found that the species richness of collembolans, earthworms, and orbatid mites was affected negatively with increasing land-use intensity due to the significant effect of food web diversity [141].

### Climate change

A long-term change in the average weather patterns or ‘climate change’ can have long-term effects on the soil microbiome and biodiversity. Temperature, CO_2_ level, and soil moisture content can be altered by climate change, thereby influencing soil biodiversity [51]. Climate change impacts the networking between the soil microbiome, by influencing its stability with different ecosystems behaving differently to each stimulus [63]; for example, under soil warming, the soil microbial populations increase by 40 -150% [63, 129].

There are various responses that soil microbiome uses to survive with the environmental conditions that are drastically changing, few of them are-

- **Elevated CO**_**2**_** [eCO**_**2**_**]**—Many studies have reported the microbiome shifts with eCO_2_ [63, 77]. A regional scale study in Australian grasslands showed that the microbial diversity [archaea, bacteria, and fungi] was significantly influenced by eCO2 over some time when exposed to [ambient + 550 ppm] concentrations of atmospheric CO2 and higher temperatures [ambient + 2 °C] [53]. Changes in functions performed by soil microbiome under eCO2 could be analyzed through quantification of the gene abundances in the metagenomes particularly genes responsible for decomposition, nitrogen fixation [63]. It is also very essential to understand how vital environmental factors like temperature, nutrients, and precipitation interacts with eCO_2_ and this can help forecast the response of soil microbiome to eCO_2_.
- **Increased temperature**- Depending upon the biome, the response of high temperature varies on the soil microbiome. For example, warming effects on soil biodiversity show weaker responses in arid ecosystems, whereas in the Arctic regions the responses are quite prominent accompanied by a significant increase in the soil populations [131]. In grassland communities, a small increment in temperature has a clear impact on soil fauna participating in root growth [108]. Studies even revealed that warming has a striking influence on the soil fungal communities under different systems, causing either suppression or stimulation of fungal biomass and its activity considering the difference in vegetation and the soil moisture [2, 3, 17, 96]. As for bacteria, members from Acidobacteria and Actinobacteria were found to be in higher proportions as compared to other bacterial phyla due to soil warming effects [123]. Although many models on climate predict that an increase in soil respiration and decrease in soil storage is found to have positive feedback as a result of warming, but is largely dependent on the ecosystems [39, 54].
- **Permafrost thaw-** Permafrost thaw of the Arctic soil is the most severe outcome of global warming. On Earth, around 20% of the terrestrial surface is occupied by permafrost soil, which is the vast reservoir of carbon that will represent the future allocation of carbon from the biosphere to the atmosphere [57]. The common feature of permafrost thaw is a change in soil moisture that largely manages the soil microbial activity [76]. Despite the indifferent conditions, permafrost is known to harbor a huge range of microbiota and especially prokaryotes and fungi with elevated functional diversity along with a huge number of unknown taxa [4, 38]. Few studies suggest that the response of microbial communities is significantly affected by the rise in temperature, which ultimately leads to change in ecosystem functioning [87]. Several metagenomic studies disclosed that the structure of the microbial community and functional capabilities in permafrost are different from those present in the active layer as compared to the permafrost microbiome that changes rapidly upon thawing [87, 104].
- **Drought**- In mesic grasslands, droughts are considered to be the main outcome of climate change [19]. The rising drought conditions are anticipating in the decreasing microbial functions, which are essential for the sustainability of ecosystems. Drought can endure high impacts on the soil microbiome in grassland ecosystems, due to the shift in vegetation to more drought-tolerant plant species and their consequent preference for various root-associated microbes [32, 105]. Both bacterial and fungal networks respond differently to drought, in which bacterial networks were observed to be strongly impacted by drought over fungal networks, which may be due to their ability to tolerate a broad range of environmental conditions [26].

## Challenges and prospects in unboxing the black box of soil microbiome

We have seen exceptional advances and insights into the understanding of the soil microbiome in the last decade, driven by improvements in the NGS technologies. This augmented understanding of soil microbiome research is accompanied by a wealth of data, which has led us to understand microbial communities like never before, including their interactions and effects in soil, with recent studies even trying to explore and predict these microbial networks at global scales [49, 88]. Despite the great progress achieved in elucidating the taxonomical diversity, their application in soil ecology remains tough. First, many of the biogeochemical processes are not the product of a single metabolic pathway but the product of multiple interconnected pathways that can be carried out by a wide range of taxa [112]. Second, the large number of individual genes like 16S and 18S rRNA and functional genes from dormant microscopic organisms may startle efforts to link functional processes to specific soil microbiome. Third, there are methodological concerns associated with linking specific taxa to particular metabolic processes; these consist of difficulties with accurate gene annotation [126], and the rapid turnover of transcripts, proteins, and metabolites [64]. Fourth, sequencing-based methodologies, which are extensively used to enumerate the abundances of taxa or functional genes in soil usually, offer mostly information about the relative abundances of taxa or genes and not their absolute abundances. Different taxa, genes, and their products are likely to be associated with different soil processes, and to examine this, different approaches are used to examine or to quantify the given set of gene responsible for that particular function [46]. And even if there is a direct relationship between the taxon and the process of interest, there is a need to understand the environmental constraints responsible for the taxa of interest. For instance, definite bacterial methane oxidizers can have different substrate affinities, in any event, when they become under controlled laboratory conditions [71].

In order to fine tune the understanding of this process, it is necessary to emphasize on each of these points result in much better understanding of the field and scope in future for numerous applications. It is attempted to narrow down approaches which could fine tune the efforts in exploring and understanding the soil microbiome efficiently.

As per the globally accepted standards for the soil microbiome field conceptualized in 2020 [5] by National Institute for Biological Standards and Control [NIBSC] for gut microbiome analysis by next-generation sequencing, this would potentially improve development of methods, reduce and check submissions of inaccurate findings, and allow for effective exchange of results among peers, globally. Furthermore, standards can open up inventions in the soil microbiome turf, as they disprove the obligation for everyone to use the same protocol as long as users corroborate their protocol with respect to the global standards [22].

Identification of interlinks between the diverse soil microbiome and their functional capabilities has always been difficult as only a small fraction of the soil-inhabiting taxa can be cultivated in the laboratories, where investigative experiments yielding detailed results can be obtained. This could be resolved by several methods like qPCR, amplicon sequencing, metagenomics, metabarcoding, and metatranscriptomics. Extensive efforts put into culturable isolation of soil microbes which are more responsive to cultivation especially from particular niches [91]. Genome sequencing and characterization of such isolates would be much easier and would provide much better insights in conjunction with the metagenomics data to answer pertinent ecological questions. Secondly, setting of simulated simplified microbiome experiments of much complex natural environments and studying them through the help of “OMICS” would yield insightful results and a better understanding of the problem. This could help us in manipulating the soil network to a lower level of complexity that is much easier to comprehend and might help us in observing the big picture [1]. This in conjunction with the metagenomic data of the original microbiome could help foresee the functional capacities of respective microbes and their role in the different bioprocesses and ultimately toward the ecosystem functioning [115, 117]. Thirdly, development of functional annotation techniques instead of amplicon based sequencing which can only determine relative abundances of taxa or genes and not their absolute abundances without any knowledge of functional profiles in the studied environments [23].

Amplicon-based sequencing of marker genes [16S rDNA, 18S rDNA, or ITS regions] have always been plagued by insufficient taxonomic resolutions due to inability of the present sequencing to sequence the whole gene. New long-read sequencing technologies like PacBio circular consensus sequencing and loop genomics synthetic long-read sequencing technology [sFL16S] can sequence the entire marker gene such as 16S rRNA gene [14, 65]. These techniques could overcome the microbial misidentification caused by previous approach’s inability to sequence the whole marker gene and could correctly classified to the sub-species clades [14, 65]. These techniques could in turn generate high quality sequences from the unculturable environmental organisms, which can then help in developing robust databases including such environmental sequences. This would eventually reduce hitches where a huge extent of bacterial and archaeal taxa found in soil have taxonomic marker gene sequences that do not match to those found in reference databases [119].

## Conclusion

There is no deficiency of information gaps that limit our comprehension of the soil microbiome and their respective roles in the soil environment. Indeed, even an answer to an inquiry as straightforward as 'what is the normal age time of soil microbes?' stays obscure. The nature, composition and complexity of soil microbiome and its interaction with other biotic and abiotic components are the key for future research applications. These applications include soil analytical studies for crop improvement, in agriculture and ecological studies for eco-restoration as well as geological studies for different types of land use pattern. There is no deficiency of information gaps that limit our comprehension of the soil microbiome and their respective roles in the soil environment. There are huge number of developing methodologies that can be and are being utilized to additionally investigate the phylogenetic and functional jobs of the soil biota. Harnessing this perspective of the soil biota and microbiome could possibly provide us with replies that can deliver us innovative solutions for present environmental challenges. Understanding the soil biodiversity at the level of taxonomic and functional attributes and its collective networking with the ecosystem functions is of utmost importance. For instance, to check the imbalances in soil and for monitoring soil quality, soil biodiversity should be considered as one of the key indicators. More efforts must be given toward developing management practices that will benefit soil biodiversity and resulting in improved restoration strategies. More specific, durable, and accurate technologies and methods are required to be developed for analyzing the soil microbiome and understanding the unknown facts about microbe–material interaction in soil.

## Supplementary Information

Below is the link to the electronic supplementary material.

E-supplementary data for this work can be found in e-version of this paper online
Supplementary Figure S1. Schematic representation of potential threats to the soil microbiomes based on recent Web of Science publications
Threats declared in 52 papers given by Web of Science with topic search threat* “soil biodiversity” on 2^nd^ December 2021. They are categorized in 5 main threats, distinguished on the basis of color code. The deeper orange shade to lighter, indicates the percentage threat to soil microbiome, starting from higher to lower threats respectively
Supplementary Table S1. List of potential metagenomic tools applied to explore soil microbiomes (TIFF 752 KB)
