# Supplementary figures and images for: Unboxing the black box—one step forward to understand the soil microbiome: A systematic review
Source: Microb Ecol. 2022 Feb 2;85(2):669–83. doi: 10.1007/s00248-022-01962-5 (PMC9957845; doi:10.1007/s00248-022-01962-5)

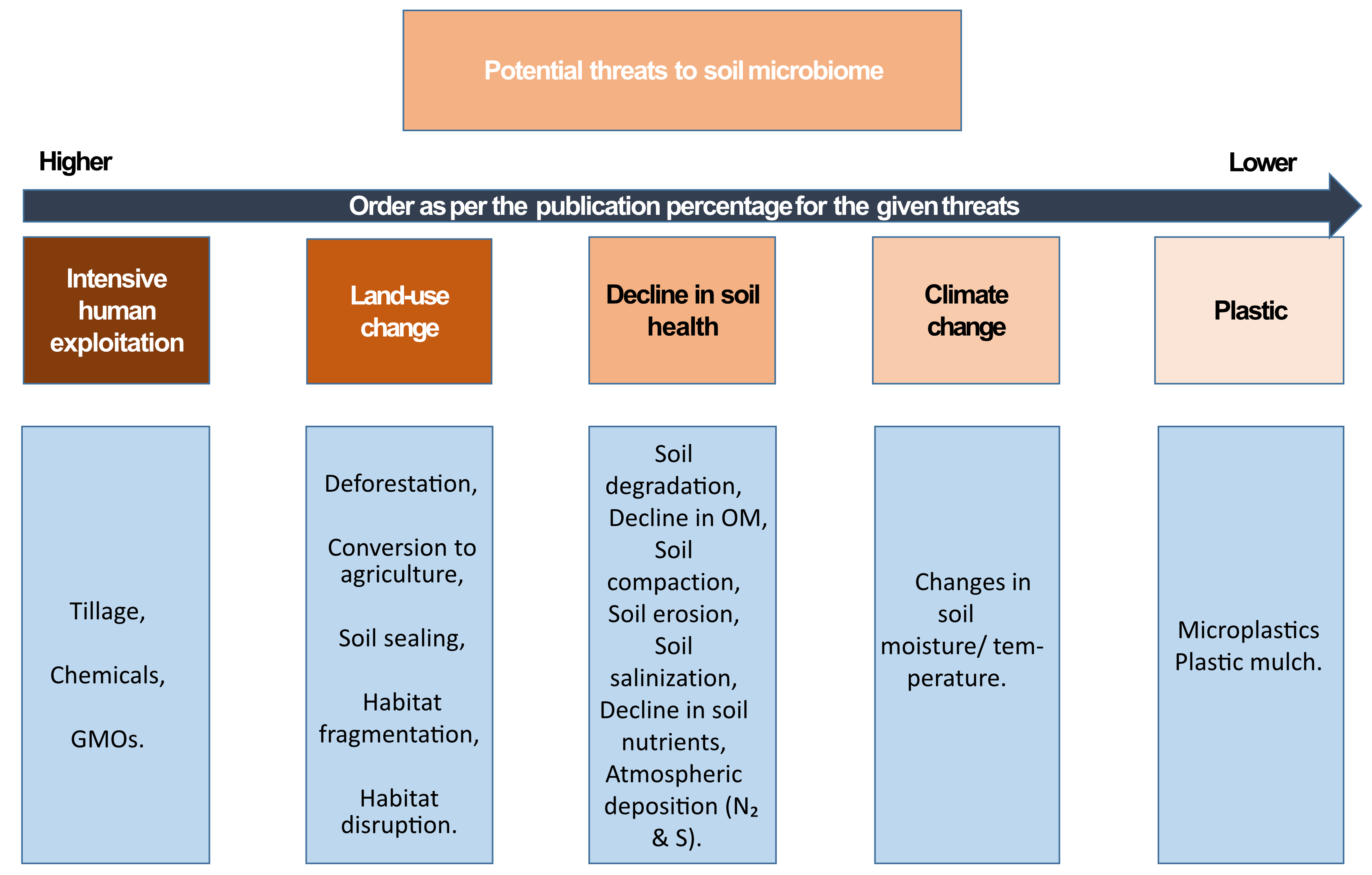

Supplement: Supplementary file 1 — E-supplementary data for this work can be found in e-version of this paper online Supplementary Figure S1. Schematic representation of potential threats to the soil microbiomes based on recent Web of Science publications Threats declared in 52 papers given by Web of Science with topic search threat* “soil biodiversity” on 2nd December 2021. They are categorized in 5 main threats, distinguished on the basis of color code. The deeper orange shade to lighter, indicates the percentage threat to soil microbiome, starting from higher to lower threats respectively Supplementary Table S1. List of potential metagenomic tools applied to explore soil microbiomes (TIFF 752 KB) [file 248_2022_1962_MOESM1_ESM.tiff]
